# The shape-dependent inhibitory effect of rhein/silver nanocomposites on porcine reproductive and respiratory syndrome virus

**DOI:** 10.1186/s11671-023-03900-x

**Published:** 2023-10-10

**Authors:** Caifeng Ren, Qiyun Ke, Xiaoxia Fan, Keke Ning, Yuan Wu, Jiangong Liang

**Affiliations:** 1 State Key Laboratory of Agricultural Microbiology, College of Resource and Environment, College of Science, Huazhong Agricultural University, Wuhan, 430070 People’s Republic of China; 2 State Key Laboratory of Agricultural Microbiology, College of Veterinary Medicine, Huazhong Agricultural University, Key Laboratory of Preventive Veterinary Medicine in Hubei Province, Cooperative Innovation Center for Sustainable Pig Production, Wuhan, 430070 People’s Republic of China; 3 College of Science, Huazhong Agricultural University, Wuhan, 430070 People’s Republic of China

**Keywords:** Porcine reproductive and respiratory syndrome virus, Rhein, Silver nanocomposites, Antiviral mechanisms, Reactive oxygen species

## Abstract

Traditional Chinese medicines (TCMs)/nanopreparations as viral antagonists exhibited a structure–function correlation, i.e., the differences in surface area/volume ratio caused by the variations in shape and size could result in different biochemical properties and biological activities, suggesting an important impact of morphology and structure on the antiviral activity of TCM-based nanoparticles. However, few studies paid attention to this aspect. Here, the effect of TCM-based nanoparticles with different morphologies on their antiviral activity was explored by synthesizing rhein/silver nanocomposites (Rhe@AgNPs) with spherical (S-Rhe/Ag) and linear (L-Rhe/Ag) morphologies, using rhein (an active TCM ingredient) as a reducing agent and taking its self-assembly advantage. Using porcine reproductive and respiratory syndrome virus (PRRSV) as a model virus, the inhibitory effects of S-Rhe/Ag and L-Rhe/Ag on PRRSV were compared. Results showed that the product morphology could be regulated by varying pH values, and both S- and L-Rhe/Ag exhibited good dispersion and stability, but with a smaller size for L-Rhe/Ag. Antiviral experiments revealed that Rhe@AgNPs could effectively inhibit PRRSV infection, but the antiviral effect was morphology-dependent. Compared with L-Rhe/Ag, S-Rhe/Ag could more effectively inactivate PRRSV in vitro and antagonize its adsorption, invasion, replication, and release stages. Mechanistic studies indicated that Rhe@AgNPs could reduce the production of reactive oxygen species (ROS) induced by PRRSV infection, and S-Rhe/Ag also had stronger ROS inhibitory effect. This work confirmed the inhibitory effect of Rhe@AgNPs with different morphologies on PRRSV and provided useful information for treating PRRSV infection with metal nanoparticles synthesized from TCM ingredients.

**Graphical abstract:**

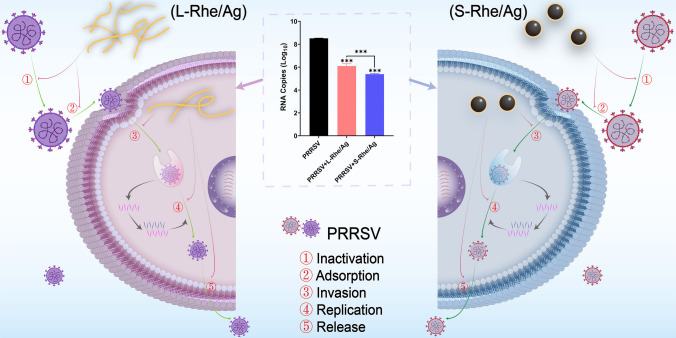

**Supplementary Information:**

The online version contains supplementary material available at 10.1186/s11671-023-03900-x.

## Introduction

Viral disease pandemics are a huge threat to global public health and economic development, while innovative antiviral drugs can contain and prevent new rounds of infections and reduce the scope of pandemics [1]. The prevention and treatment of viral diseases is a hot research subject worldwide, because the existing antiviral drugs suffer the defects such as poor solubility, short cycle half-life, and low bioavailability, thus limiting their efficacy [2]. In response to these defects, nanomaterials have been shown to contribute to the development of new drugs and biologics due to their outstanding optical, chemical and biological properties, displaying great potential in the field of antiviral research. Studies have shown that the efficacy of a range of antiviral materials can be improved by nanotechnology [2], where nanomaterials have been engineered for virus prevention, diagnosis, and treatment, and successfully used in preclinical models. Among them, functional metal nanoparticles have been reported as effective antiviral drugs [3]. Introducing metal nanoparticles into antiviral therapy offers unique advantages and a unique mechanism, where the sizes, morphologies, surface chemistries, and charges of nanoparticles are demonstrated to affect their antiviral activities [4]. For example, only nanoparticles in the 1–10 nm range were found able to bind to sulfur-containing residues on the viral envelope. Mori et al. studied the effect of silver nanoparticles (Ag NPs) with three particle sizes (3.5 nm, 6.5 nm, and 12.9 nm) on H1N1 influenza A virus and found that Ag NPs with a smaller particle size had stronger antiviral activity [5]. Morphology has also been reported to affect antiviral activity. For instance, EI-Sheekh et al. prepared shape- and size-tunable Ag_2_O-AgO NPs (spherical) and Au NPs (octahedral, pentagonal, and triangular) through green synthesis and found that the antiviral activity against HSV was slightly higher in Ag_2_O-AgO NPs than in Au NPs [6]. Additionally, Sinclair et al. investigated the effect of capping agents with surface charges ranging from negative to positive on the anti-MS2 phage activity of Ag NPs and found that electrostatic interactions between cationic capping agents and the negatively charged virus surface could bring the virus closer to the toxic silver particles, enabling lower and more efficient dosing of Ag NPs in antiviral applications due to the synergistic effect of Ag and capping agent [7]. Studies have also indicated that surface charge can affect the toxicity of curcumin nanoparticles (Cur NPs) to alveolar macrophages, and positively charged Cur NPs exhibited higher anti-inflammatory activity than negatively charged and neutral Cur NPs [8]. The synthetic methods of metal nanoparticles are very diverse, but they can be programmatically modified to control size, charge, and morphology to increase antiviral activity and reduce cytotoxicity.

Among the many synthetic methods of metal nanoparticles, biosynthesis has the advantages of being cleaner, non-toxic, and environmentally friendly [9]. Currently, various metal nanoparticles have been successfully synthesized from different components of plants, thus broadening the application prospect of antiviral nanotechnology [10–12]. For example, gold nanoparticles (Au NPs) synthesized with *garlic* extract as reducing agent have been reported as effective virus inactivators for measles virus [13], and the particle size of Au NPs is around 11 nm but not sufficiently dispersed. Some researchers have synthesized zinc oxide nanoparticles (ZnO NPs) with fresh *mentha spicata leaf* extract, which could effectively act on tobacco mosaic virus, but the particle sizes of the synthesized ZnO NPs were extremely heterogeneous, in a distribution range of 11–80 nm, and their morphology is also both spherical and lumpy, leading to their poor dispersion [14]. Additionally, the fruit extract of *Syzygium alternifolium (Wt.) Walp.* was used to synthesize copper oxide nanoparticles (CuO NPs), which were shown to have strong inhibitory effect against Newcastle disease virus, but their particle size distribution was in the range of 2–69 nm, indicating poor homogeneity [15]. These reports suggest that selection of plant components and optimization of synthesis methods are potential factors limiting the application of metal nanoparticles in the antiviral field.

Medicinal plants (i.e., traditional Chinese medicine, TCM) have unique advantages in anti-inflammatory, antiviral, and antibacterial properties [16], and TCM has been recorded to have achieved remarkable results in the control and treatment of infectious diseases over the past 3000 years [17]. The latest direct evidence is that in the early stage of COVID-19 outbreak, TCM played an important role in the effective control of this epidemic in China. There are 8 kinds of TCMs commonly used in the anti-epidemic stage, which were reported to contain 12 main active ingredients [18]. Traditional Chinese medicine has the advantages of relatively low economic cost, easy collection, and fewer side effects, thus favoring its promotion in the antiviral field [19]. However, TCM still faces the problems of large dosage, slow efficacy, poor biocompatibility, and the need for continuous administration [16]. This suggested that using traditional Chinese medicine components as reducing agents or capping agents for synthesis of metal nanoparticles is a promising strategy for developing new antiviral drugs, which can from a synergistic effect, better exert the TCM efficacy, and solve its limitations in clinical application. Silver-based nanomaterials are one of the most widely studied metal nanomaterials. In recent years, medicinal plant molecules have been successfully used as functionalized ligands in the green synthesis of Ag NPs [20]. The synergistic effect of silver-based materials and TCM has many typical successful cases in the antiviral field. For instance, Ag NPs modified with tannic acid, ~ 24 nm in size, were shown to effectively kill herpes simplex virus 2 and exert systemic protection [21]. Additionally, Sharma’s research group studied the antiviral activity of Ag NPs modified by *Phyllanthus niruri*, *Andrographis paniculata*, and *Tinospora cordifolia* against chikungunya virus, whose particle sizes were in the range of 70–120 nm, 70–95 nm, and 50–70 nm, respectively, and *Andrographis paniculata*-modified Ag NPs in the size range of 70–95 nm were found able to inhibit the virus activity to the greatest extent [22]. Moreover, Sreekanth et al. tried to synthesize Ag NPs with *ginseng root* extract, and by adjusting the molar ratio of silver, Ag NPs in the particle size range of 5–15 nm were obtained, which showed obvious killing effect on influenza A viruses (IAV) in a silver molar ratio-dependent manner [23]. Furthermore, silver nanoparticles synthesized by medicinal plants were considered to have a place in the future antiviral field, and their antiviral effect was reported to be size-dependent [24]. However, the relationship between their morphology and antiviral activity remains to be explored.

PRRSV, an enveloped single-stranded positive-stranded RNA virus with at least 10 open reading frames (ORFs) in its genome [25–27], is highly contagious and can cause a series of clinical symptoms such as high fever and diarrhea, leading to huge economic losses to the global pig industry [28]. The current prevention and control program mainly adopts strict biosafety measures and immunization, but the virus is prone to mutation and recombination, producing mixed infection and resulting in a low protection rate of existing vaccines. At present, there is no effective prevention and control strategy to block the occurrence and transmission of the disease [26], suggesting the necessity to develop new and more effective anti-PRRSV drugs.

Studies have confirmed that many TCMs have multi-channel anti-PRRSV effects in vitro [16, 29], and rhein, a TCM, is an anthraquinone derivative with the ability to inhibit the activity of various viruses [30–32]. Rhein also has an environmental response mechanism and can self-assemble into hydrogels through non-covalent interactions such as π–π stacking and hydrogen bonding [33]. The advantages of the self-assembly of rhein with organic–inorganic carriers can reduce the potential toxicity of nanoparticles [34]. However, rhein is almost insoluble in water, and the poor water solubility limits its clinical application. Therefore, this study intended to use rhein in the synthesis of metal nanocomposites to solve its problem of water solubility, enabling the synergistic effect of rhein and silver as well as the synthesis of rhein/silver nanocomposites (Rhe@AgNPs) with different shapes for the research of their inhibitory effects on PRRSV. This study provided a method for preparing traditional Chinese medicine-based Ag NPs with different morphologies against PRRSV infection.

## Experimental part

### Synthesis of Rhe@AgNPs

Rhe@AgNPs were synthesized using a previous one-pot method with some modifications [3]. Briefly, rhein solution (20 mM) was mixed with 22.5 mL of deionized water, followed by adjusting pH to alkaline with 0.1 M potassium carbonate solution (pH = 7.2–9.2), and then transferring the mixture to the oil bath. After stirring continuously, 2.5 mL of silver nitrate solution was added, and the reaction was continued in a 100 °C oil bath for 1 h. After cooling to room temperature, based on the molecular weight of the reactants, the large precipitates were removed by centrifugation, followed by dialysis for 5 h with a dialysis bag at a molecular weight cut-off of 1000 Da (with deionized water changed once in the middle of dialysis). Finally, the solution was collected and freeze-dried at − 30 °C for 48 h to obtain spherical rhein/silver nanocomposites (S-Rhe/Ag) and linear rhein/silver nanocomposites (L-Rhe/Ag).

### Cytotoxicity assay

MARC-145 cells were seeded in 96-well plates and cultured until about 80–90% confluence. After discarding the supernatant, Rhe@AgNPs (S- and L-Rhe/Ag) were added separately to the cell culture. At the same time, a group of cells was treated with Dulbecco’s modified eagle medium (DMEM) medium containing 2% fetal bovine serum (FBS) and used as a control. After incubation for 12, 24, 36, and 48 h, the cell viability was assessed using the MTT assay [35, 36].

### Preparation of antiviral assay samples

To evaluate the antiviral effect of the materials, MARC-145 cells were incubated with different concentrations (0.0, 4.0, 8.0, and 16.0 µg/mL) of Rhe@AgNPs in an incubator for 2 h. Meanwhile, PRRSV strains at a different multiplicity of infection (MOI) (0.1, 0.01, 0.001, and 0.0001 MOI) were incubated on ice with different concentrations of Rhe@AgNPs for 1 h. After adding the above viral material mixture into the cell plate and incubation for 1 h, the supernatant was discarded, followed by two washes with sterile phosphate buffered saline (PBS) and then incubation with the corresponding concentrations of S- and L-Rhe/Ag. Finally, cell samples were collected at the indicated time points for indirect immunofluorescence assay (IFA), real-time quantitative reverse transcription polymerase chain reaction (RT-qPCR), and Western blot assays [37, 38].

### Inactivation assay

RT-qPCR was used to investigate whether Rhe@AgNPs could directly inactivate PRRSV. Briefly, PRRSV (MOI = 0.01) was incubated with S- and L-Rhe/Ag (8.0 µg/mL and 16.0 µg/mL, respectively) at 37 °C for 1 h. At the same time, the monolayer cell plates were pre-cooled at 4 °C for 30 min. Afterward, pretreated PRRSV was added and incubated at 4 °C for 2 h [39], followed by collecting the samples with TRIzol for RT-qPCR.

### Adsorption assay

RT-qPCR assay was used to investigate whether Rhe@AgNPs could inhibit the adsorption process of PRRSV. Briefly, the monolayer cell plates were pre-cooled at 4 °C for 30 min, followed by incubating PRRSV (MOI = 0.01) with S-Rhe/Ag or L-Rhe/Ag (8.0 µg/mL and 16.0 µg/mL, respectively) for 2 h [40], and then collecting the samples with TRIzol for RT-qPCR.

### Invasion assay

RT-qPCR was used to investigate whether Rhe@AgNPs could inhibit the invasion process of PRRSV infection. Briefly, monolayer cell plates were pre-cooled at 4 °C for 30 min, followed by infection with PRRSV (MOI = 0.01) at 4 °C for another 2 h. Finally, the infected cells were incubated separately with 8.0 µg/mL and 16.0 µg/mL of S- and L-Rhe/Ag at 37 °C for 3 h [41] and then collected with TRIzol for RT-qPCR.

### Replication assay

RT-qPCR was used to investigate whether Rhe@AgNPs could inhibit the replication process of PRRSV. Briefly, monolayer cell plates were infected with PRRSV (MOI = 0.01) for 1 h in an incubator at 37 °C and further cultured with fresh DMEM (containing 2% fetal bovine serum) for 6 h. Finally, the cells were incubated separately with S- and L-Rhe/Ag (8.0 µg/mL and 16.0 µg/mL) for 7, 8, 9, and 10 h and then collected with TRIzol for RT-qPCR [42].

### Release assay

TCID_50_ assays were used to investigate whether Rhe@AgNPs could inhibit the release process of PRRSV. Briefly, monolayer cell plates were infected with PRRSV (MOI = 0.01) for 1 h in an incubator at 37 °C and further cultured with fresh DMEM (containing 2% fetal bovine serum) for another 17 h. Then, Rhe@AgNPs (S- and L-Rhe/Ag) with a concentration of 8.0 µg/mL and 16.0 µg/mL were added to the 24-well plate and cultured for 15, 30, 45, and 60 min, respectively. Finally, the supernatant was collected [43], and the virus content in the corresponding samples was determined by the TCID_50_ assay [41].

Detailed information on the reagents, cells, viruses and biological experiments used in the experiments, as well as information on the equipment used for all the characterizations involved in this study, are provided in Additional file 1.

## Results

### Characterization and analysis of Rhe@AgNPs

Based on the characteristic that the self-assembly degree of rhein can be regulated by pH, the Rhe@AgNPs were prepared under different pH conditions, and their morphology and structure were characterized by transmission electron microscopy (TEM) (Additional file 1: Fig. S1). In Fig. S1, it can be observed that at pH = 7.2, 7.6 and 8.0, rhein plays the role of reducing agent during self-assembly reactions, resulting in linear Rhe@AgNPs (hereinafter, L-Rhe/Ag). With increasing alkalinity, the cross-linking degree of the gel was enhanced (Fig. S1a–c), consistent with the previous report that the cross-linking degree of rhein varies with pH [33]. The UV–Vis absorption spectra under the above three pH conditions are shown in Fig. S1d–f, where all of them were seen to exhibit the characteristic absorption peak of Ag at around 420 nm. The nanoparticles size gradually increased with the increase in pH, causing a slight shift in the Ag absorption peak position with the change of particle size, agreeing with the previous result [44]. Fully spherical Rhe@AgNPs (hereinafter, S-Rhe/Ag) were obtained at pH > 8.0 (pH = 8.4, 8.8, 9.2). Along with the enhancement of basicity, S-Rhe/Ag showed further improvement in dispersion and uniformity (Fig. S1g–i). The corresponding UV–Vis absorption spectra are shown in Fig. S1j–l, where the nanocomposites size was seen to further increase with the increase in pH, causing the characteristic absorption peak position of Ag to be still affected by the particle size. The above results showed the successful synthesis of Rhe@AgNPs with linear and spherical morphologies (L- and S-Rhe/Ag). Based on the dispersibility of nanocomposites and the cross-linking degree of the gel, the cross-linking degree in the gel state was optimal at pH = 8.0 (Fig. S1c), and the nanocomposites exhibited the optimal uniformity and dispersibility at pH = 9.2 (Fig. S1i), so the materials under the two reaction conditions of pH = 8.0 and pH = 9.2 were selected for subsequent experiments. The surface morphology and size distribution of Rhe@AgNPs at pH = 8.0 and pH = 9.2 were analyzed by high-resolution transmission electron microscopy (HR-TEM).

**Fig. 1:**
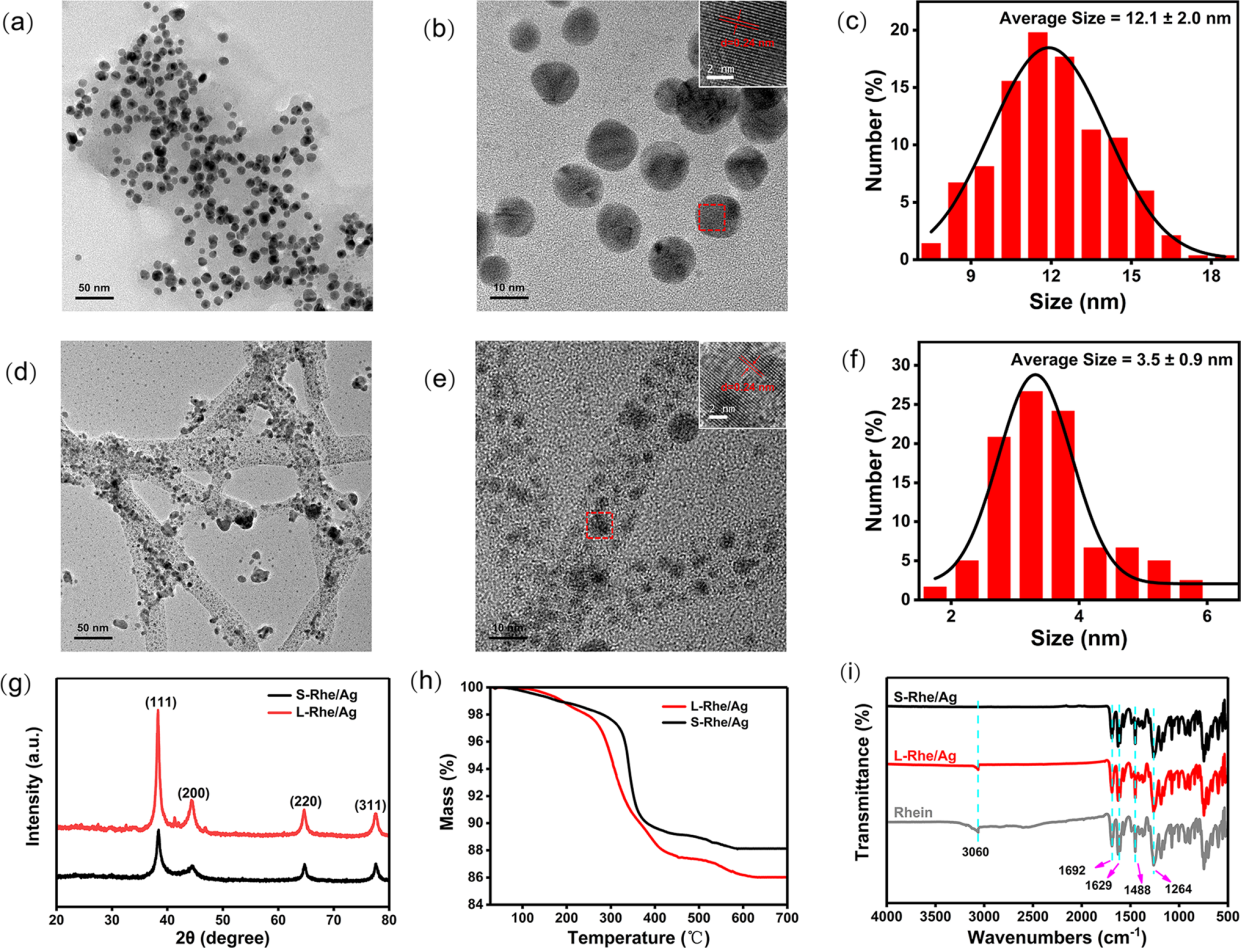
Characterization of Rhe@AgNPs. **a–c** The TEM image, HR-TEM image, and particle size distribution histogram of S-Rhe/Ag, respectively. **d–f** The TEM image, HR-TEM image, and particle size distribution histogram of L-Rhe/Ag, respectively. **g** XRD patterns of S-Rhe/Ag and L-Rhe/Ag. **h** Thermogravimetric analysis results of S-Rhe/Ag and L-Rhe/Ag. **i** Fourier-transform infrared spectra (FTIR) of S-Rhe/Ag, L-Rhe/Ag, and rhein

In Fig. 1a, S-Rhe/Ag were seen to be in the form of spherical particles, with uniform dispersion and particle size distribution. The lattice structure of S-Rhe/Ag was seen to have obvious lattice fringes with a lattice size of 0.24 nm, and by measuring 100 particles, the average particle size of S-Rhe/Ag was 12.1 nm (Fig. 1b–c). As shown in Fig. 1d–f, L-Rhe/Ag were linear, the particles inside were round and uniformly distributed, and the lattice fringes were clearly visible, with a size of 0.24 nm. The average diameter of L-Rhe/Ag is about 15 nm, and by measuring 100 particles, the average particle size of L-Rhe/Ag is 3.5 nm, which is much smaller than that of S-Rhe/Ag (12.1 nm). This is probably because the rhein in the reaction system of L-Rhe/Ag first self-assembled to form a gel when the pH value was low, which could play a reducing effect to form nanoparticles with the increase in reaction time. The optical and chemical property results in the figures further demonstrate the successful preparation of Rhe@AgNPs. The maximum intensity crystalline morphology of Rhe@AgNPs could be observed in the X-ray diffraction results (Fig. 1g), where S-Rhe/Ag and L-Rhe/Ag corresponded to the crystal surface reflections of (111), (200), (220), and (311) planar cubic structures at 2θ = 38.2°, 44.4°, 64.7°, and 77.7° [38], and the face-cubic structure of L-Rhe/Ag indicated that they were distributed in the gel [45]. The FTIR spectra (Fig. 1i) can reflect the composition of Rhe@AgNPs, and compared with rhein, both S- and L-Rhe/Ag were seen to retain 5 absorption peaks near 3060 cm^−1^, 1692 cm^−1^, 1629 cm^−1^, 1488 cm^−1^, and 1264 cm^−1^, corresponding to 4 chemical bonds (O–H, C=O, C=C, and C–O bond) [34, 46]. The absorption peaks of L-Rhe/Ag are slightly shifted, indicating the involvement of hydrogen bonding in their self-assembly process [33]. The characteristic peak of S-Rhe/Ag at 3060 cm^−1^ disappeared, indicating that the original intramolecular hydrogen bond (O–H) of rhein was destroyed and O–Ag bond was successfully formed. The specific gravity of rhein in the synthesized Rhe@AgNPs was investigated by thermogravimetric analysis (TGA). In Fig. 1h, S-Rhe/Ag were seen to degrade at 307 °C, with a total mass loss rate of 88.11% at 582 °C, in contrast to a total mass loss of 13.98% for L-Rhe/Ag at 625 °C.

**Fig. 2:**
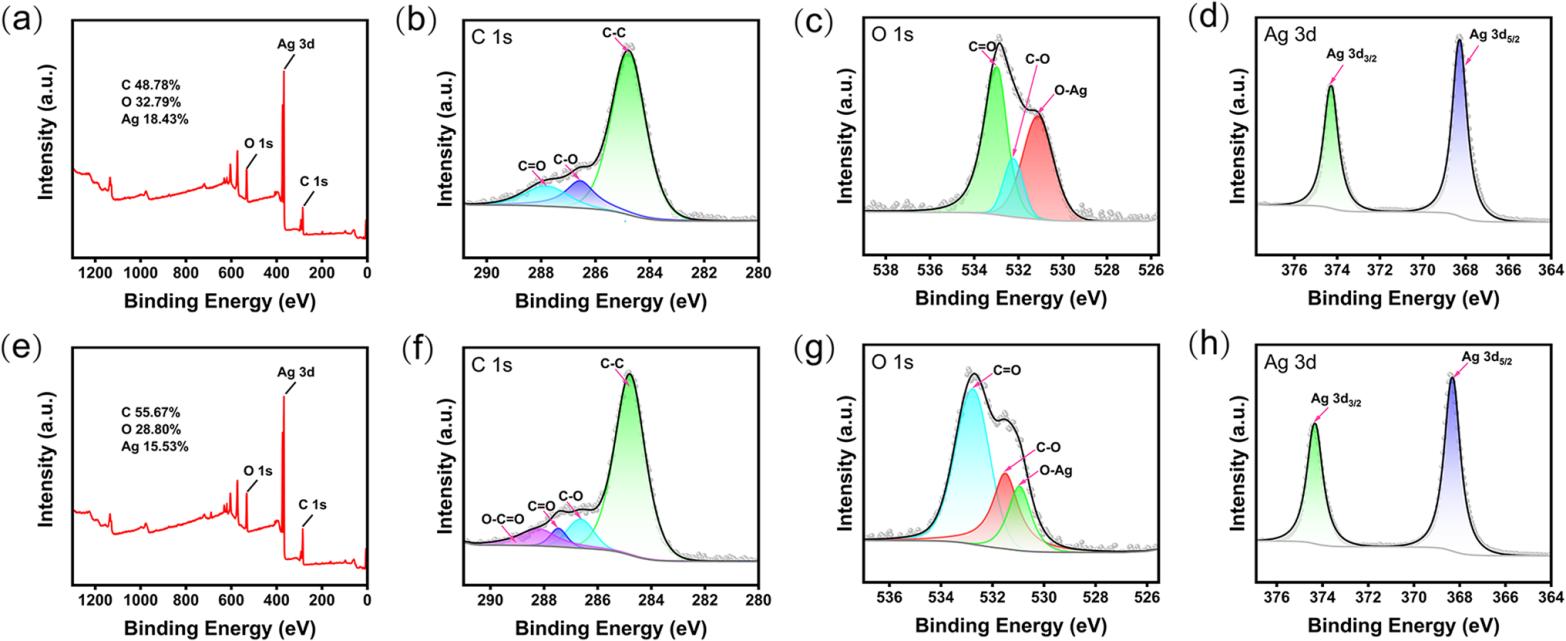
X-ray photoelectron spectra (XPS) of Rhe@AgNPs. **a** Full-scan spectra of S-Rhe/Ag and high-resolution XPS spectra of **b** C 1 s, **c** O 1 s, and **d** Ag 3d. **e** Full-scan spectra of L-Rhe/Ag and high-resolution XPS spectra of **f** C 1 s, **g** O 1 s, and **h** Ag 3d

The surface functional groups of Rhe@AgNPs were analyzed by X-ray photoelectron spectroscopy (XPS). In Fig. 2a and e, the full-range XPS analysis results showed that S- and L-Rhe/Ag had 3 distinct peaks at binding energies of 532.30 eV, 368.31 eV, and 285.10 eV, corresponding to O 1 s, Ag 3d, and C 1 s orbitals, respectively. Elemental analysis indicated that S- and L-Rhe/Ag had a carbon content of 48.78% and 55.67%, an oxygen content of 32.79% and 28.80%, and a silver content of 18.43% and 15.53%, respectively. The difference in silver content is consistent with the TGA results. The high-resolution XPS spectra of C 1 s showed that L-Rhe/Ag had one more peak at 288.16 eV than S-Rhe/Ag (Fig. 2b and f), which belonged to the O–C=O bond. The high-resolution XPS spectra of Ag 3d (Fig. 2d and h) confirmed the existence of Ag, and the binding energies of 368.26 eV and 374.27 eV corresponded to the peaks at Ag 3d_5/2_ and Ag 3d_3/2_, respectively [47]. The high-resolution XPS spectra of O 1 s of L- and S-Rhe/Ag can be fitted to three peaks, with the peaks at 532.96 eV, 532.23 eV, and 531.12 eV for S-Rhe/Ag, and the peaks at 532.76 eV, 531.50 eV, and 530.95 eV for L-Rhe/Ag, corresponding to C=O, C–O, and O–Ag bond [48]. The larger peak area at 531.12 eV in S-Rhe/Ag indicated they had a higher silver element content (Fig. 2c and g), agreeing with the elemental analysis results.

### Cytotoxicity of S-Rhe/Ag and L-Rhe/Ag

In order to compare the biocompatibility of S- and L-Rhe/Ag, and determine their safe concentration range, the cytotoxicity of S- and L-Rhe/Ag was detected. MTT assays showed that the cell viability was above 85% under the treatment conditions with a concentration of less than 32.0 μg/mL, and both S- and L-Rhe/Ag had no obvious toxicity to MARC-145 cells (Additional file 1: Fig. S2). The negligible cytotoxicity of S- and L-Rhe/Ag may be attributed to the high biocompatibility of rhein [49] and the small size of these two materials. As previously reported, the cytotoxicity of silver nanoparticles was size-dependent, and smaller particle size showed less toxicity [50]. In this study, the subsequent antiviral experiments were performed separately using 4.0 μg/mL, 8.0 μg/mL and 16.0 μg/mL Rhe@AgNPs, and using 0.0 μg/mL as the control.

### Inhibitory effects of S-Rhe/Ag and L-Rhe/Ag on PRRSV

**Fig. 3:**
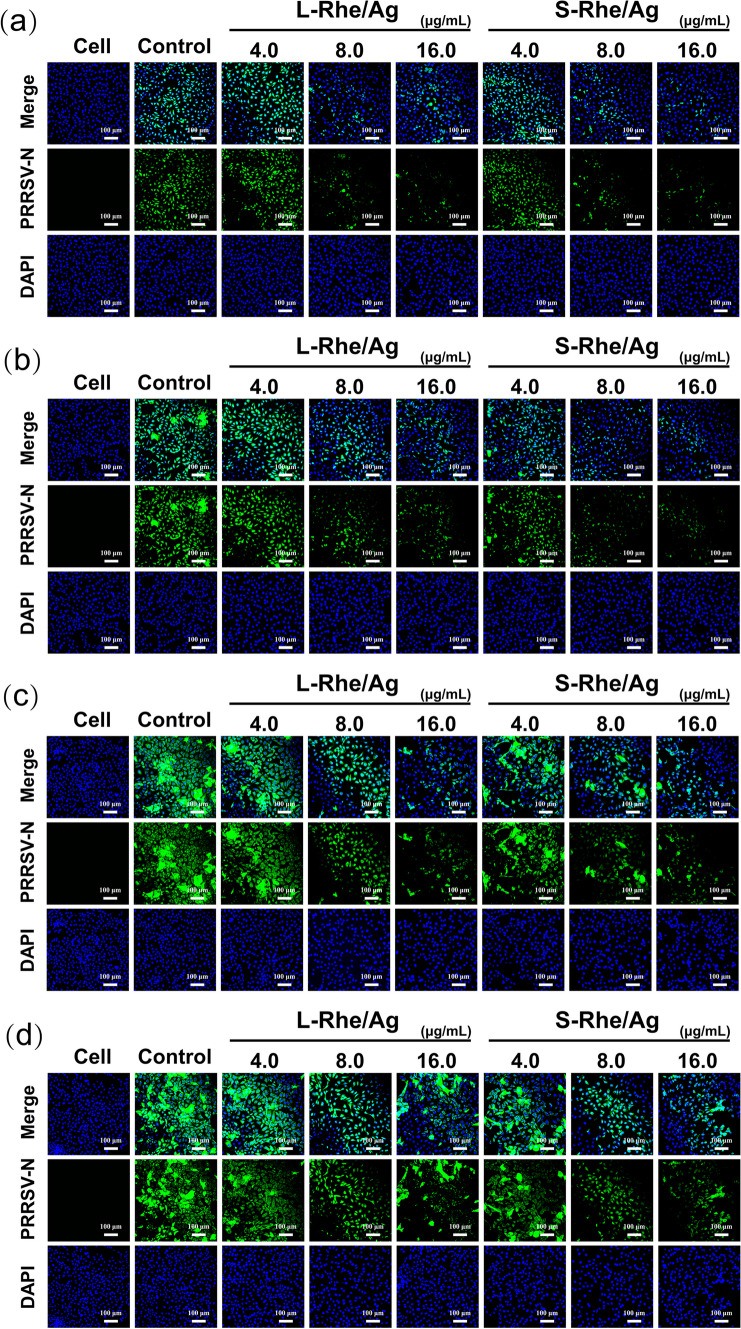
Indirect immunofluorescence images of PRRSV-infected cells treated separately with S-Rhe/Ag or L-Rhe/Ag for 12 h (**a**), 24 h (**b**), 36 h (**c**), and 48 h (**d**). Blue represents the nuclei, and green represents the PRRSV N protein, with all the pictures as randomly selected regions in the field of view. The control group was only infected with PRRSV and untreated with Rhe@AgNPs

The antiviral effects of both silver nanoparticles and rhein have been previously reported [30, 51], but whether the composite of the two possesses antiviral activity has not been investigated. Here, PRRSV, an economically important virus that has been devastating the pig industry worldwide, was used as a virus model to explore the potential antiviral effects of S- and L-Rhe/Ag. Firstly, their concentration and time gradient effects on PRRSV proliferation were evaluated by IFA. In Fig. 3, the blue fluorescent signals indicate the nucleus, and the green fluorescent signals represent the PRRSV N protein labeled with N protein-specific mouse monoclonal antibody and Alexa Fluor® 488-conjugated donkey anti-mouse IgG. At each time period, the green fluorescence signal decreased in a dose-dependent manner, indicating that S- and L-Rhe/Ag had anti-PRRSV activity. The antiviral activity of Ag NPs is strongly particle size- and morphology-dependent, and Ag NPs with a size of 10 nm were reported to better interact with viruses [52]. Consistent with previous findings, S-Rhe/Ag with a particle size of 12 nm exhibited stronger inhibitory effect on PRRSV infection than L-Rhe/Ag with a particle size of 3.5 nm, probably due to the easier access of spherical materials to the interior of cells.

**Fig. 4:**
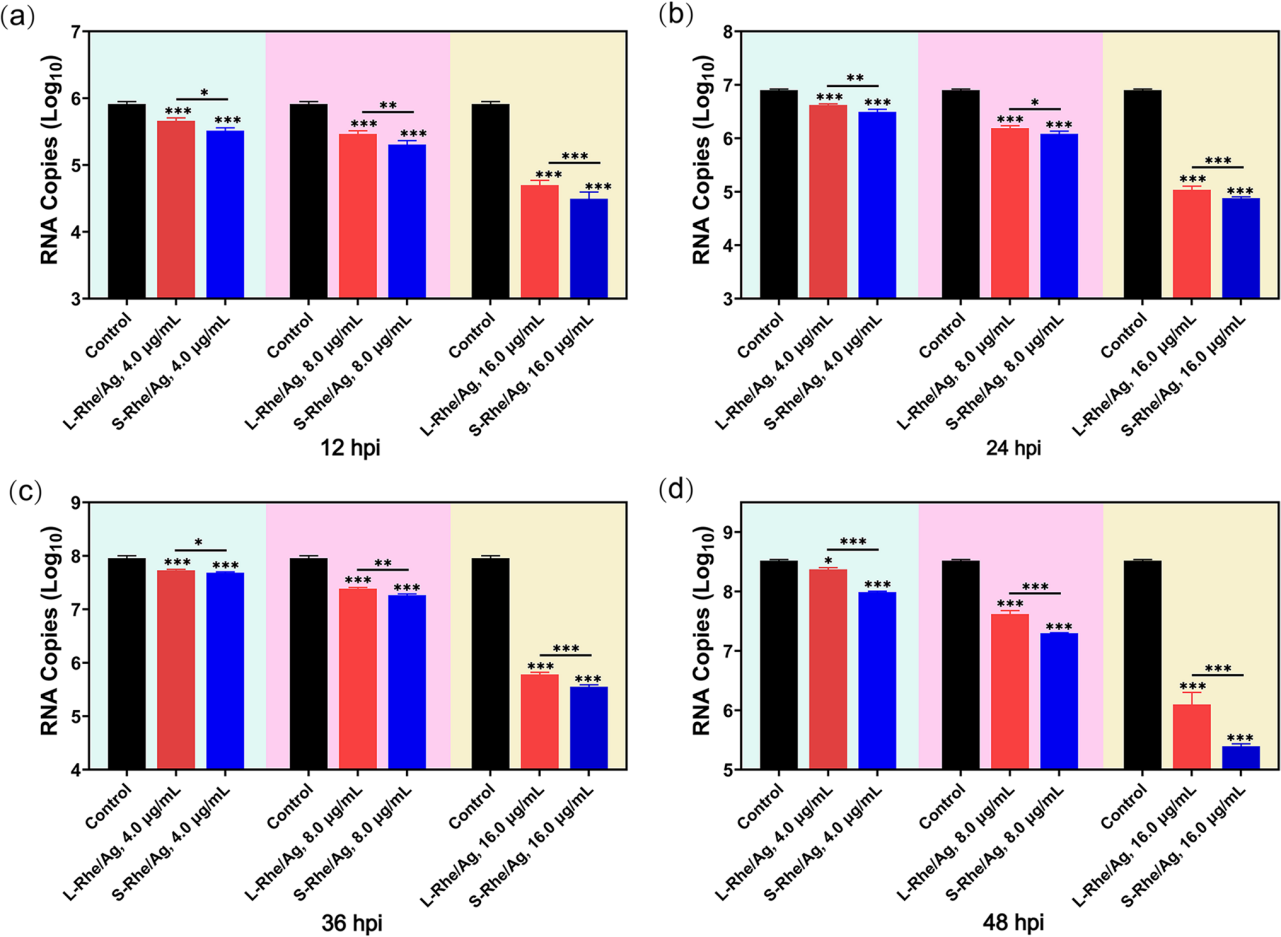
The antiviral effects of Rhe@AgNPs on PRRSV infection evaluated by RT-qPCR. The content of PRRSV ORF7 gene in MARC-145 cells treated with S-Rhe/Ag or L-Rhe/Ag (0.0–16.0 μg/mL) for 12 h (**a**), 24 h (**b**), 36 h (**c**), and 48 h (**d**). MARC-145 cells in the control group were only infected with PRRSV and untreated with Rhe@AgNPs. Error bars represent the standard deviation from three repeated experiments. *p < 0.05, **p < 0.01, and ***p < 0.001 indicate a significant difference relative to the indicated groups

**Fig. 5:**
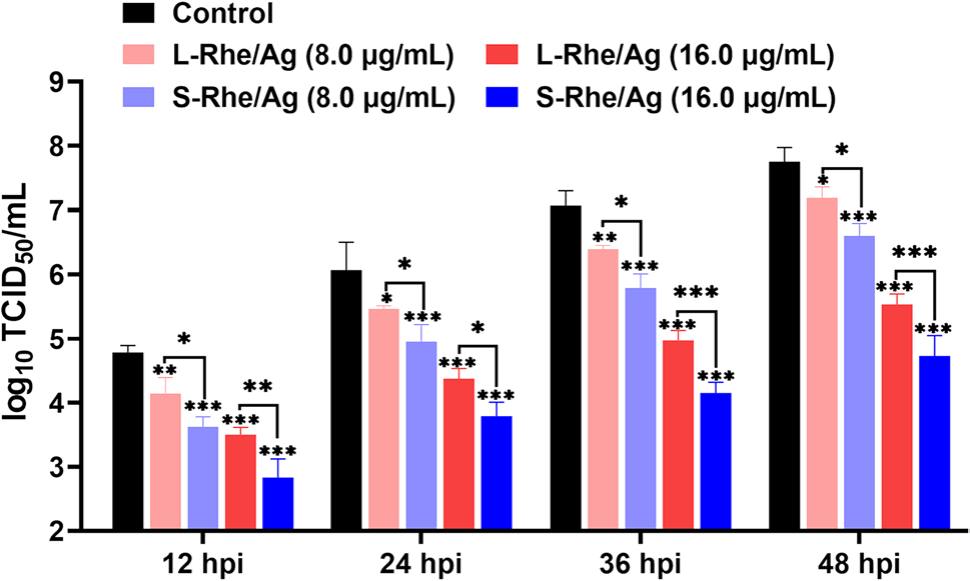
Detection of the difference between S-Rhe/Ag and L-Rhe/Ag in inhibitory effects on PRRSV by TCID_50_ assays. MARC-145 cells in the control group were only treated with PRRSV infection. Error bars represent the standard deviation from three repeated experiments. *p < 0.05, **p < 0.01, and ***p < 0.001 indicate a significant difference relative to the indicated groups

In order to further verify the difference between S-Rhe/Ag and L-Rhe/Ag in inhibiting PRRSV proliferation, RT-qPCR was performed to detect the RNA expression level of PRRSV ORF7. As shown in Fig. 4, both S-Rhe/Ag and L-Rhe/Ag dose dependently decreased the content of PRRSV ORF7 gene. At the concentration of 16.0 μg/mL, S- and L-Rhe/Ag significantly reduced the virus titers in the cells by about 2 log. Meanwhile, S-Rhe/Ag were shown to be significantly stronger than L-Rhe/Ag in the inhibitory effects on PRRSV ORF7 gene levels at each time point. The above results are consistent with the previous report that the antiviral efficacy of TCM-based Ag NPs was concentration- and time-dependent [53]. Additionally, TCID_50_ assays were also performed to confirm the above conclusion. As shown in Fig. 5, in each time period, both S- and L-Rhe/Ag had remarkable inhibitory effects on the PRRSV titers, but S-Rhe/Ag exhibited significantly stronger inhibitory effects than S-Rhe/Ag, consistent with the RT-qPCR and IFA results, which was also related to the influence of morphology and size on anti-PRRSV effect.

**Fig. 6:**
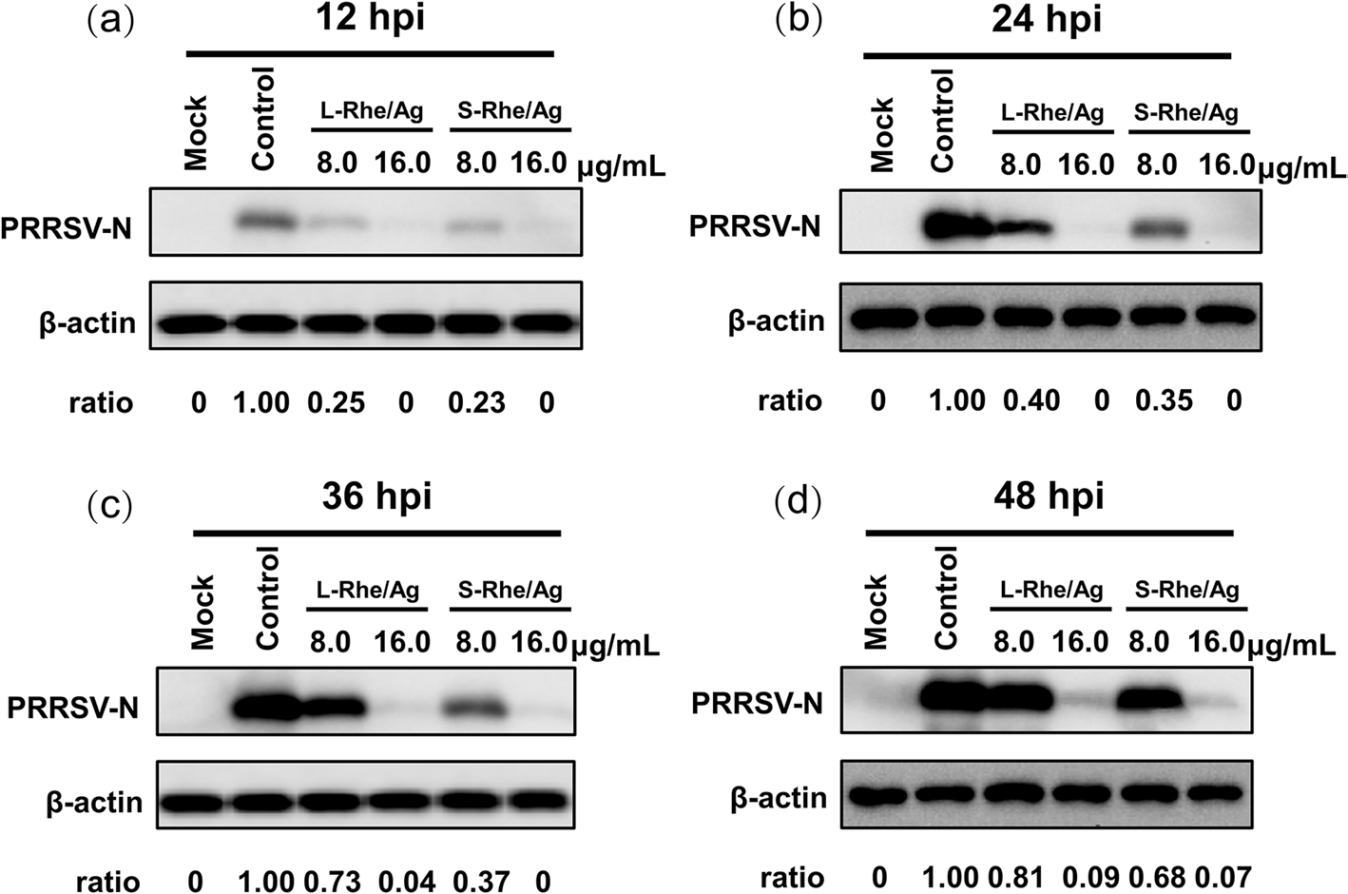
Western blot analysis of the difference between S-Rhe/Ag and L-Rhe/Ag in their inhibitory effect on PRRSV at the nucleocapsid protein level after incubation at 8.0 μg/mL and 16.0 μg/mL concentrations for 12 h (**a**), 24 h (**b**), 36 h (**c**), and 48 h (**d**), respectively

The nucleocapsid (N) protein of PRRSV plays a key role in virus replication. The expression level of PRRSV N protein was analyzed by immunoblotting to further explore the difference between S-Rhe/Ag and L-Rhe/Ag in inhibiting PRRSV proliferation. In Fig. 6 and Additional file 2, the expression level of N protein was seen to decrease significantly after incubation with Rhe@AgNPs, revealing the inhibitory effect of both Rhe@AgNPs on PRRSV proliferation, but S-Rhe/Ag exhibited higher inhibitory intensity than L-Rhe/Ag, agreeing with the RT-qPCR results.

**Fig. 7:**
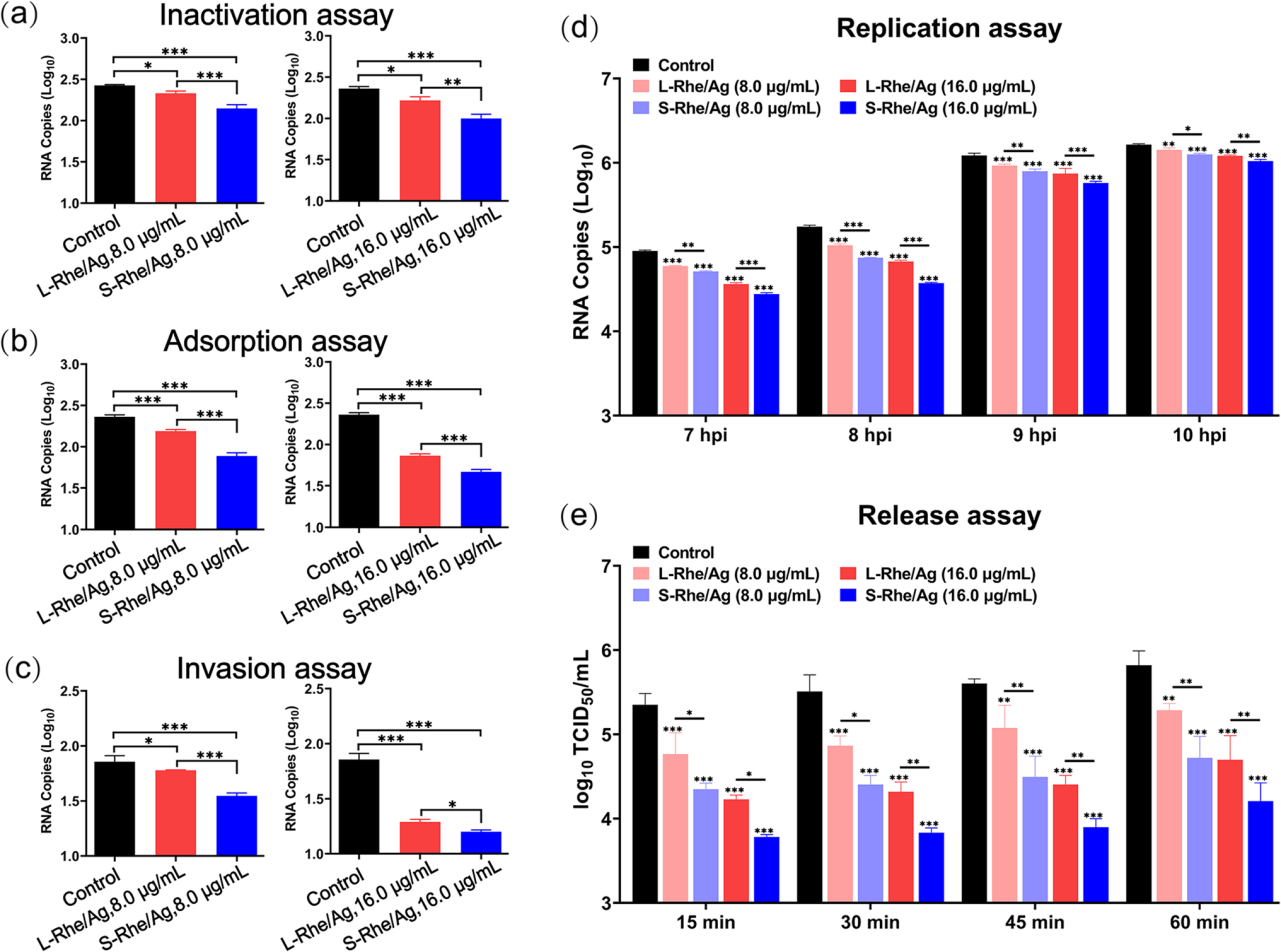
Multistage inhibitory effects of Rhe@AgNPs on PRRSV proliferation. **a** Inactivation of PRRSV by S-Rhe/Ag and L-Rhe/Ag. Effects of S-Rhe/Ag and L-Rhe/Ag on PRRSV adsorption (**b**), invasion (**c**), replication (**d**), and release (**e**). Error bars represent the standard deviation from three repeated experiments. MARC-145 cells in the control group were only treated with PRRSV infection. *p < 0.05, **p < 0.01, and ***p < 0.001 indicate a significant difference relative to the indicated groups

The potential inhibitory mechanism of S- and L-Rhe/Ag on PRRSV was investigated by analyzing their direct inactivation effect on the proliferation cycle of PRRSV at incubation concentrations of 8.0 μg/mL and 16.0 μg/mL. In a previous study, administration of rhein alone was found unable to inactivate IAV [30], but our inactivation assay results showed a significant dose-dependent decrease in virus levels (Fig. 7a), indicating that Rhe@AgNPs had direct inactivation effects, and S-Rhe/Ag exhibited stronger virucidal activity. This finding reveals more inhibitory sites in Rhe@AgNPs than in the active ingredients of traditional Chinese medicine administered alone, highlighting the advantage of their synergistic effect.

Following virus infection, viral proliferation involves several typical stages, with a potential drug target for each stage. Based on PRRSV life cycle, the effects of S- and L-Rhe/Ag on PRRSV proliferation at the four stages were further explored. When PRRSV enters cells, the replication process begins under the guidance of the positive-sense genomic RNA, and quantifying the level of negative-sense RNA in the viral genome can effectively evaluate the effect of Rhe@AgNPs on the replication process. After replication is completed, PRRSV will be released outside the cell to continue the infection of the next cell to start a new reproduction cycle, so the effective inhibition of the release process can also prevent further infection of the virus. Figure 7b–e shows the inhibitory effects of S- and L-Rhe/Ag on the life cycle of PRRSV at 8.0 μg/mL and 16.0 μg/mL. Both S- and L-Rhe/Ag could reduce the RNA level of PRRSV at each stage, but the viral RNA level decreased more significantly after S-Rhe/Ag treatment, indicating that S-Rhe/Ag could more effectively impede the process of PRRSV adsorption, invasion, replication, and release than L-Rhe/Ag.

### Rhe@AgNPs inhibit ROS production in PRRSV-infected MARC-145 cells

Reactive oxygen radicals (ROS) are accessory substances of cellular metabolism [54]. Some viruses have been reported to cause the release of intracellular ROS, stimulate oxidative stress in cells, promote viral replication [55, 56], and ultimately lead to impaired cellular organization, indicating ROS is a potential antiviral target [54]. High doses of Ag NPs were shown to induce ROS production, thereby blocking multiple related oxidative stress signaling pathways [57]. Studies have indicated that silver nanoparticle-based oseltamivir administration can reduce ROS levels and thus have anti-H1N1 virus activity. Inflammation plays an important role in PRRSV infection and pathogenic mechanisms, and ROS is a marker of pathogenic oxidative damage in inflamed tissues and is also the main cause of oxidative stress [58]. This suggested the necessity to investigate whether Rhe@AgNPs may also down-regulate ROS levels to achieve anti-PRRSV activity.

**Fig. 8:**
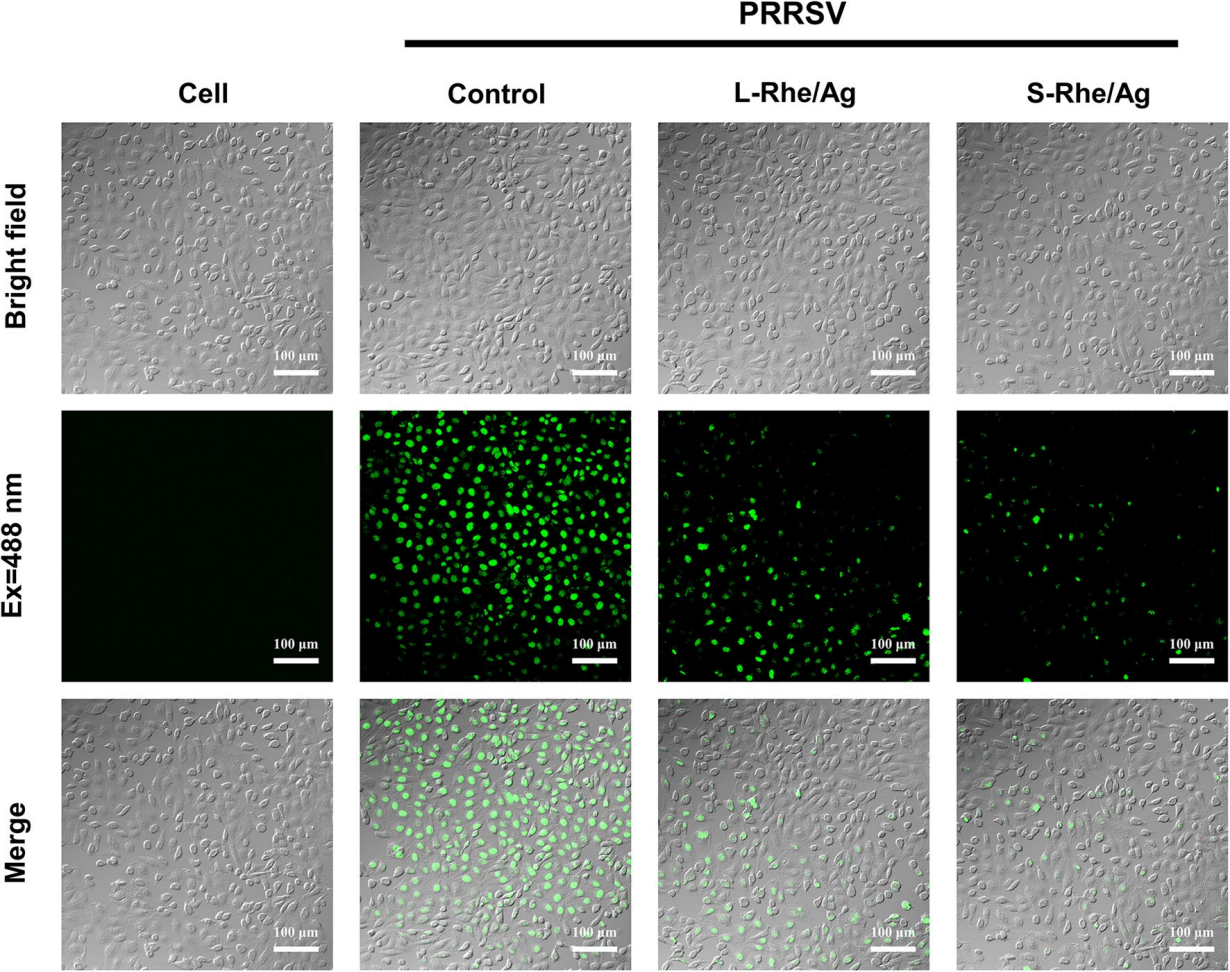
DCFH-DA detection of the effect of Rhe@AgNPs on PRRSV-induced ROS production. Cell group, PRRSV group, L-Rhe/Ag group, and S-Rhe/Ag group represent no treatment, treatment only with PRRSV infection, co-treatment of PRRSV and L-Rhe/Ag, and co-treatment of PRRSV and S-Rhe/Ag, respectively

The ROS levels were measured with the fluorescent probe DCFH-DA on MARC-145 cells, and the results are shown in Fig. 8. The green fluorescence signal, which represents the ROS amount, was seen to be weaker in the Rhe@AgNPs-treated group than in the control group (infected with PRRSV only). Meanwhile, the fluorescence signal was weaker in the S-Rhe/Ag-treated group than in the L-Rhe/Ag-treated group, indicating that Rhe@AgNPs could also interfere with the ROS level induced by PRRSV infection, and S-Rhe/Ag had a stronger inhibitory effect on ROS.

## Discussion

TCMs have attracted increasing attention due to their superior pharmacological activities, including antiviral, antibacterial, anti-inflammatory and antitumor effects. In recent years, anti-PRRSV TCMs have also been widely reported [42, 59–62]. However, as antiviral drugs, the disadvantages of TCMs such as a long medication cycle and poor water solubility have limited their development and application. Through nanotechnology and special drug delivery systems, all kinds of TCMs can break through the limitations of traditional medication methods and improve their economic and medicinal value. The designed drug delivery systems of TCM can be divided into two types: carrier and carrier-free [63]. The active ingredients of TCM, alkaloids, can improve efficacy while reducing harmful effects through specially designed drug delivery systems (liposome specific delivery, nanoparticle sustained delivery, etc.). For example, the intravenous injection of chitosan-encapsulated berberine nanoparticles (CS NPs) into mice could prolong the residence time and efficacy of TCM components in vivo [64]. Through carrier-free delivery, the active component curcumin could be engineered into carbon dots to make it an excellent antiviral agent against enterovirus 71 (EV 71), and the cytotoxicity and antiviral capacity of the engineered curcumin carbon dots were 1,000-fold lower and 34-fold higher than that of curcumin, respectively [65]. Some TCM active molecules have self-assembling properties, enabling them to be more targeted, and have higher bioavailability potential in drug delivery systems [66]. Studies have shown that Ag NPs are one of the most popular research materials, which can exert different antiviral mechanisms and have broad-spectrum antiviral properties [39, 67–71]. Ag NPs have also been reported to possess anti-PRRSV effect and are potential anti-PRRSV drugs [72], so the present study evaluated the inhibitory effect of TCM-based Ag NPs on PRRSV.

In recent years, researchers have combined silver and certain herbal components in different ways. One is the direct use of various parts of the plant body by extracting chemicals with organic solvents, but this extraction method is inefficient, causing the resulting Ag NPs to have a wide size distribution, poor monodispersity, and varying morphology [22]; the second method is the use of TCM active ingredients to prepare Ag NPs. For instance, Orlowski et al. [73] prepared three sizes of spherical Ag NPs in the presence of tannic acid and sodium citrate by modulating the amount of silver nitrate during synthesis, and the Ag NPs prepared under this condition had only a single morphology. Rhein can self-assemble to form hydrogels through non-covalent forces, with the self-assembly degree being regulated by pH [33], allowing it to act as a reducing agent during biosynthesis of Ag NPs. The morphology and size of Ag NPs are affected by the pH value of solution, reducing agent, synthesis method, and other conditions [74]. The use of rhein’s self-assembly behavior in synthesis of Ag NPs could lead to stable structures with host–guest interactions [75, 76]. Based on this, we explored the physicochemical properties of Rhe@AgNPs under different pH conditions. By changing pH, we successfully prepared two Rhe@AgNPs with different morphologies: S-Rhe/Ag and L-Rhe/Ag, both of which are highly soluble and monodisperse in water. The average particle size of as-prepared Rhe@AgNPs was less than 15 nm, which was much smaller than the size of PRRSV virus [26], showing low cytotoxicity. At the same time, the self-assembly degree of L-Rhe/Ag increased with increasing pH with pH ≤ 8.0, due to the slow reduction rate of the precursor in this pH range, where the self-assembly process of rhein mainly occurs. The self-assembly results are consistent with the previous report that pH could significantly affect the self-assembly process of rhein, while silver introduced in rhein makes L-Rhe/Ag more stable by forming a host–guest structure [33, 77]. However, when pH > 8.0, both the dispersibility and size of the product S-Rhe/Ag were enhanced with the increase in pH, probably due to the rapid reduction of the precursor at a high pH value [78].

Metal nanoparticles have multi-target effects on the antiviral mechanism of PRRSV and can act on all stages of virus proliferation [36, 37]. In addition, the shape and size of nanoparticles may lead to differences in antiviral activity. Orlowski et al. [73] used the same dose of tannic acid and conventional sodium citrate as a reducing agent in the preparation of Ag NPs, which may not highlight the medicinal properties of tannic acid, making the obtained Ag NPs unable to inactivate the viruses, and the efficiency of virus inhibition was better only at a size of 13 nm. In this study, both L-Rhe/Ag and S-Rhe/Ag exhibited good biocompatibility and potent antiviral activity, inactivating PRRSV in vitro and exerting inhibitory activity in multiple stages of PRRSV proliferation (adsorption, invasion, replication, and release). However, unlike L-Rhe/Ag, S-Rhe/Ag exhibited stronger inhibitory effect on PRRSV proliferation and ROS produced by the virus, thus a better anti-PRRSV advantage than L-Rhe/Ag, indicating that the morphology of nanosilver is a key factor affecting the antiviral efficacy [79]. This study demonstrates that the introduction of TCM into silver-based nanomaterials can serve as a strategy for developing anti-PRRSV drugs.

Active ingredients in TCMs have less adverse effects on the human body and are often cost-effective in terms of productivity [80], making them potential lead compounds. Silver-based nanomaterials can effectively inactivate RNA and DNA viruses in vitro [81]. In the present study, Rhe@AgNPs were green synthesized with rhein, a TCM component, which is simple to operate and can well inhibit the proliferation of PRRSV and has the potential as an environmentally friendly antiviral agent. However, the synthesis and storage of Rhe@AgNPs still need more consideration due to the stability of self-assembly of TCM active ingredients. The cytotoxicity of silver-based nanomaterials is a limiting factor for their development, due to their interaction with different types of cells in a complex and varied manner [4], so the antiviral efficacy of L-Rhe/Ag and S-Rhe/Ag in vivo and clinical applications needs to be further verified. In addition, the synthesis method can lead to various sizes of silver-based nanomaterials of plant components, which in turn can affect the cytotoxicity and the action effect [82], so it is necessary to explore new and effective synthesis methods. At the same time, the pharmacological effects of TCMs are complex and diverse, enabling them to have the effect of enhancing the body’s resistance [83], and Ag NPs can play an immunomodulatory role by regulating pro-inflammatory and chemokines, implicating that potential antiviral strategy of Rhe@AgNPs can be further revealed in the future [84].

## Conclusion

In this study, by taking advantage of its self-assembly properties, the TCM rhein was used as a raw material to prepare rhein-modified silver nanocomposites (Rhe@AgNPs), which showed spherical (S-Rhe/Ag) and linear (L-Rhe/Ag) shapes by varying pH values, integrating the mechanisms of viral inhibition by rhein and silver-based nanomaterials, enabling both of them to exert inhibitory effects in the early-, middle-, and late stages of PRRSV proliferation. Importantly, S-Rhe/Ag had a stronger anti-PRRSV effect than L-Rhe/Ag as influenced by morphology. This study demonstrated the synergistic effect of nanocarriers and TCM, revealing novel clues for the antiviral effects of TCM mixtures, and providing a basis for the use of TCM for PRRSV prevention and treatment.

### Supplementary Information

**Additional file 1.** Includes details of reagents, cells and viruses used in the experiment, equipment used for all characterizations (UV-Vis, FTIR, TEM, XRD, TGA) involved in this study, experimental procedures for MTT assay, indirect immunofluorescence assay, western blotting assay, real-time quantitative reverse transcription polymerase chain reaction, virus titration and ROS assay, TEM images and UV-Vis absorption spectra of L-Rhe/Ag and S-Rhe/Ag at differect pH, cytotoxicity of L-Rhe/Ag and S-Rhe/Ag on MARC-145 cells.

**Additional file 2.** Includes all the unprocessed gel and blot diagrams which is to corroborate the authenticity of the western blotting assay results in the manuscript.

## Data Availability

The data used to support this review are included within the article.

## Code availability

Source code is available upon request from the corresponding author.
