# Supplementary material for: The shape-dependent inhibitory effect of rhein/silver nanocomposites on porcine reproductive and respiratory syndrome virus
Source: Discov Nano. 2023 Oct 10;18(1):126. doi: 10.1186/s11671-023-03900-x (PMC10564707; doi:10.1186/s11671-023-03900-x)
Supplement: Supplementary file 1 — Additional file 1. Includes details of reagents, cells and viruses used in the experiment, equipment used for all characterizations (UV-Vis, FTIR, TEM, XRD, TGA) involved in this study, experimental procedures for MTT assay, indirect immunofluorescence assay, western blotting assay, real-time quantitative reverse transcription polymerase chain reaction, virus titration and ROS assay, TEM images and UV-Vis absorption spectra of L-Rhe/Ag and S-Rhe/Ag at differect pH, cytotoxicity of L-Rhe/Ag and S-Rhe/Ag on MARC-145 cells. [file 11671_2023_3900_MOESM1_ESM.docx]

**Supplementary Information**

The shape-dependent inhibitory effect of rhein/silver nanocomposites on porcine reproductive and respiratory syndrome virus

Caifeng Ren1^‡^, Qiyun Ke^2‡^, Xiaoxia Fan^1^, Keke Ning^3^, Yuan Wu^3^, Jiangong Liang^1^*

^1^ State Key Laboratory of Agricultural Microbiology, College of Resource and Environment, College of Science, Huazhong Agricultural University, Wuhan 430070, P. R. China.

^2^ State Key Laboratory of Agricultural Microbiology, College of Veterinary Medicine, Huazhong Agricultural University, Key Laboratory of Preventive Veterinary Medicine in Hubei Province, Cooperative Innovation Center for Sustainable Pig Production, Wuhan 430070, P. R. China.

^3^ College of Science, Huazhong Agricultural University, Wuhan 430070, P. R. China.

‡ Caifeng Ren and Qiyun Ke contributed equally to this paper.

*Correspondence: liangjg@mail.hzau.edu.cn

**Author information：**

Caifeng Ren: rencf@webmail.hzau.edu.cn

Qiyun Ke: keqiyun@webmail.hzau.edu.cn

Xiaoxia Fan: 1714484336@qq.com

Keke Ning: queenkerrning@outlook.com

Yuan Wu: yuanwu@mail.hzau.edu.cn

Jiangong Liang: liangjg@mail.hzau.edu.cn

Experimental methods

**Chemicals and Reagents**

Rhein (AR, ≥97%) was provided by Aladdin Chemical Co., Ltd. (Shanghai). Potassium carbonate (K_2_CO_3_, AR, ≥98%), silver nitrate (AgNO_3_, AR, ≥99%) and dimethyl sulfoxide (DMSO, CP, ≥ 98%) were all obtained from Sinopharm Chemical Reagent Co., Ltd. (Shanghai). All reagents were used directly without further purification. All experimental water was provided by Millipore ultrapure water purification system (Milli-Q, Millipore, 18.25 MΩ). DMEM medium was purchased from Hyclone and fetal bovine serum (FBS) was purchased from Gibco. Ampicillin, kanamycin, and trypsin were purchased from Invitrogen. DAPI, mouse monoclonal antibody and Alexa Fluor® 488-conjugated donkey anti-mouse IgG were purchased from AntGene Co., Ltd (USA).

**Viruses and Cell Culture**

Monkey kidney (MARC-145) cells were purchased separately from the American Type Culture Collection (ATCC) and the China Center for Type Culture Collection (CCTCC). Cells were cultured in Dulbecco’s modified Eagle’s medium (DMEM) supplemented with 10% fetal bovine serum (FBS) and 1% penicillin/streptomycin at 37 °С in a humidified CO_2_ incubator. When grown to confluence, the cells were washed with PBS and collected from the culture vessel surface by adding 0.06% trypsin.

The PRRSV strain WUH3 (GenBank Accession No. HM853673) (isolated at the end of 2006 in China from the pig brains with the syndrome of “high fever”) was transfected into MARC-145 cells as previously reported [1].

**Characterization of Rhe@AgNPs**

UV-Vis absorption spectra were recorded from 200 nm to 600 nm on a Shimadzu UV-2450 spectrometer (Shimadzu, Japan) equipped with a 10 mm quartz cell. The FTIR spectra were recorded with a Thermo Fisher Nicolet Avatar-330 infrared spectrometer (Thermo, USA) and scanned between 4000 and 500 cm^−1^. TEM images were obtained by a H-7650 transmission electron microscope (HITACHI, Japan). HR-TEM images were obtained by a JEM-2100F transmission electron microscope (JEOL Japan). The X-ray diffraction (XRD) was conducted with a D8 Advance X-ray Diffractometer (BRUKER, Germany) with Cu K_α_ radiation at 40 mA and 40 kV. The scanning of the diffraction angle (2θ) was from 20° to 80° with a scanning speed of 10° min^-1^. XPS scanning was performed on a Thermo Fisher ESCALAB Xi X-ray photoelectron spectrometer (Thermo, USA) to obtain the surface elemental composition and chemical state of the sample. Thermogravimetric analysis (TGA) was performed with a DSC200PC analyzer (NETZSCH, Germany). The gene content was detected by Real time fluorescence quantitative PCR (RT-qPCR) instrument Quant Studio 7 (ABI, USA). The indirect immunofluorescence results and ROS analysis results were obtained by Laser confocal microscope LSM880 (ZEISS, Germany).

**Cell viability was assessed by MTT assay**

MARC-145 cells were seeded in 96-well plates to a confluence of approximately 80-90%, followed by incubation separately with Rhe-AgNPs, and hRhe-AgNPs at different concentrations in DMEM supplemented with 2% FBS for 12, 24,36, and 48 h. After replacing the supernatant with 100 µL of fresh DMEM (2% FBS), each well was supplemented with 20 µL of MTT (3- [4,5- dimethylthiazol-2-thiazolyl]-2,5-diphenyl tetrazolium bromide, Sigma) solution (5.0 mg/mL). After incubation for 4 h, the supernatant was removed, and the formazan crystals were dissolved in 150 µL per well of dimethyl sulfoxide (DMSO). After shaking on a rocking shaker for 10 min at 150 rpm, the OD values at 570 nm were measured for estimating the percentage of cell relative viability using a microplate reader.

**Indirect immunofluorescence assay (****IFA)**

According to the above test sample preparation steps, samples were collected after the cells were incubated with the material for 12, 24, 36 and 48 h (all operations were performed at room temperature). First, the cells were washed three times with pre-cooled PBS (5 min each time), then fixed with 4% paraformaldehyde at room temperature for 15 min, and treated with pre-cooled methanol for 10 min, washed with PBS for three times (5 min each time), and then sealed with 5% BSA for 45 min. Then, the primary antibody (anti-PRRSV N protein mouse monoclonal antibody) was added for 1 h. After the primary antibody was recovered, the secondary antibody (Alexa Fluor 488-conjugated donkey anti-mouse IgG) was added for 1 h in the dark, and then the nucleus was treated with DAPI [2]. After staining for 15 min, imaging analysis was performed under a laser confocal microscope.

**Western blotting assay (Western-blot)**

According to the preparation steps of the antiviral test samples, the samples were collected after the cells were incubated with the material for 12, 24, 36 and 48 h. First wash 3 times with pre-cooled PBS, add lysate and let it act on ice for 10 min, then use a pipette to blow down the cells repeatedly, transfer them to a 1.5 mL EP tube, and centrifuge at 4 °C/12000 rpm for 10 minutes. After centrifugation, the supernatant was taken, added with 5×SDS-PAGE protein loading buffer, boiled in boiling water for 10 min, and then stored at -20 °C for later use. The protein samples were then subjected to gel electrophoresis to visualize the expression level of the PRRSV nucleocapsid protein (N) to reveal the infection intensity of the virus [3].

**Real-time quantitative reverse transcription polymerase chain reaction (RT-qPCR)**

According to the preparation steps of the antiviral test samples, the samples were collected after the cells were incubated with the material for 12, 24, 36h and 48 h. Briefly, pre-chilled TRIzol were added, and the cells were blown off and sorted into 1.5mL EP tubes. Using the characteristic that the ORF7 gene content of PRRSV is proportional to the infection intensity and the number of infected cells, the inhibitory ability of the material was analyzed by detecting the content of ORF7.

**Virus titration**

MARC-145 cells were seeded into 96-well plates for 12 h before infection, then cells were infected by 100 μL per well of serial 10-fold diluted supernatant in quintuplicate. Count the number of lesions after 4 days of infection, and the 50% tissue culture-infected dose (TCID_50_) was calculated by the Reed-Muench method.

**Evaluation of ROS production**

According to the preparation steps of antiviral test samples, after the cells were incubated with S-Rhe/Ag and L-Rhe/Ag for 12 h, the residual material was washed with PBS. Add 1 mL of 2',7'-dichlorofluorescein diacetate (DCF-DA) (5 µM) to each well of the cell plate and incubate in the dark for 30 min in the incubator. The staining solution was removed by washing three times with PBS, and the fluorescence images were observed by confocal microscopy to assess the level of ROS production [4].

**Statistical Analysis**

Computational analysis was performed using GraphPad Prism software. Experimental data were expressed as mean ± standard deviation, and t-test was used to analyze significance.


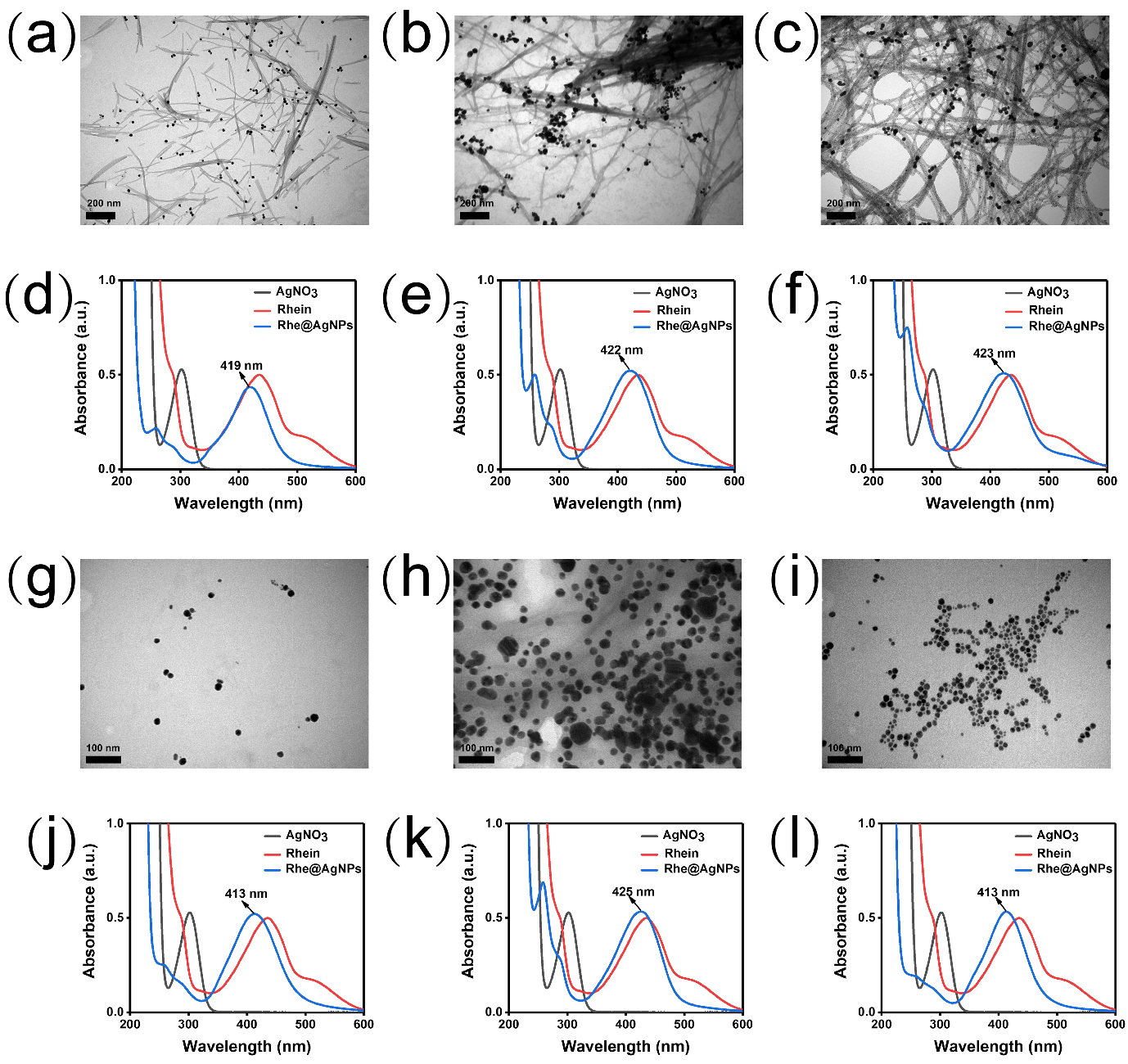


**Fig. S1** Characterization of Rhe@AgNPs under different pH conditions. When pH≤8.0 (pH=7.2, 7.6, 8.0), the products were all L-Rhe/Ag, corresponding TEM images (**a-c**) and UV-Vis absorption spectra (**d-f**). When pH>8.0 (pH=8.4, 8.8, 9.2), the products were all S-Rhe/Ag, the corresponding TEM images (**g-i**) and UV-Vis absorption spectra (**j-l**)


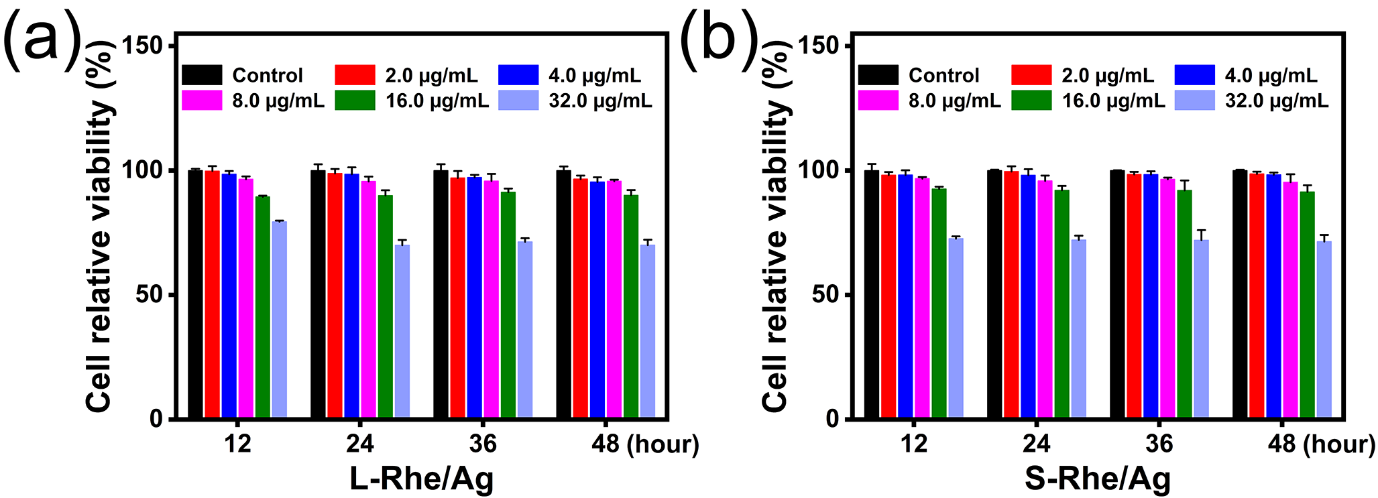


**Fig. S2** Cytotoxicity of Rhe@AgNPs on MARC-145 cells. (**a**) Cells were cultured with different concentrations of L-Rhe/Ag (0-32.0 μg/mL) for 12, 24, 36 and 48 h. (**b**) Cells were cultured with different concentrations of S-Rhe/Ag (0-32.0 μg/mL) for 12, 24, 36 and 48 h

**References**

1. Li B, Fang LR, Guo XL, Gao JF, Song T, Bi J, He KW, Chen HC, Xiao SB. Epidemiology and evolutionary characteristics of the porcine reproductive and respiratory syndrome virus in china between 2006 and 2010. J Clin Microbiol. 2011;49:3175-83.

2. Du T, Zhang JY, Li CQ, Song T, Li P, Liu JF, Du XJ, Wang S. Gold/silver hybrid nanoparticles with enduring inhibition of coronavirus multiplication through multisite mechanisms. Bioconjugate Chem. 2020;31:2553-63.

3. Du T, Liang JG, Dong N, Lu J, Fu YY, Fang LR, Xiao SB, Han HY. Glutathione-capped Ag_2_S nanoclusters inhibit coronavirus proliferation through blockage of viral RNA synthesis and budding. ACS Appl Mater Interfaces. 2018;10:4369-78.

4. Tong T, Hu HW, Zhou JW, Deng SF, Zhang XT, Tang WT, Fang LR, Xiao SB, Liang JG. Glycyrrhizic-acid-based carbon dots with high antiviral activity by multisite inhibition mechanisms. Small. 2020;16:e1906206.
