# Supplementary material for: The shape-dependent inhibitory effect of rhein/silver nanocomposites on porcine reproductive and respiratory syndrome virus
Source: Discov Nano. 2023 Oct 10;18(1):126. doi: 10.1186/s11671-023-03900-x (PMC10564707; doi:10.1186/s11671-023-03900-x)
Supplement: Supplementary file 2 — Additional file 2. Includes all the unprocessed gel and blot diagrams which is to corroborate the authenticity of the western blotting assay results in the manuscript. [file 11671_2023_3900_MOESM2_ESM.pptx]

## Slide 1
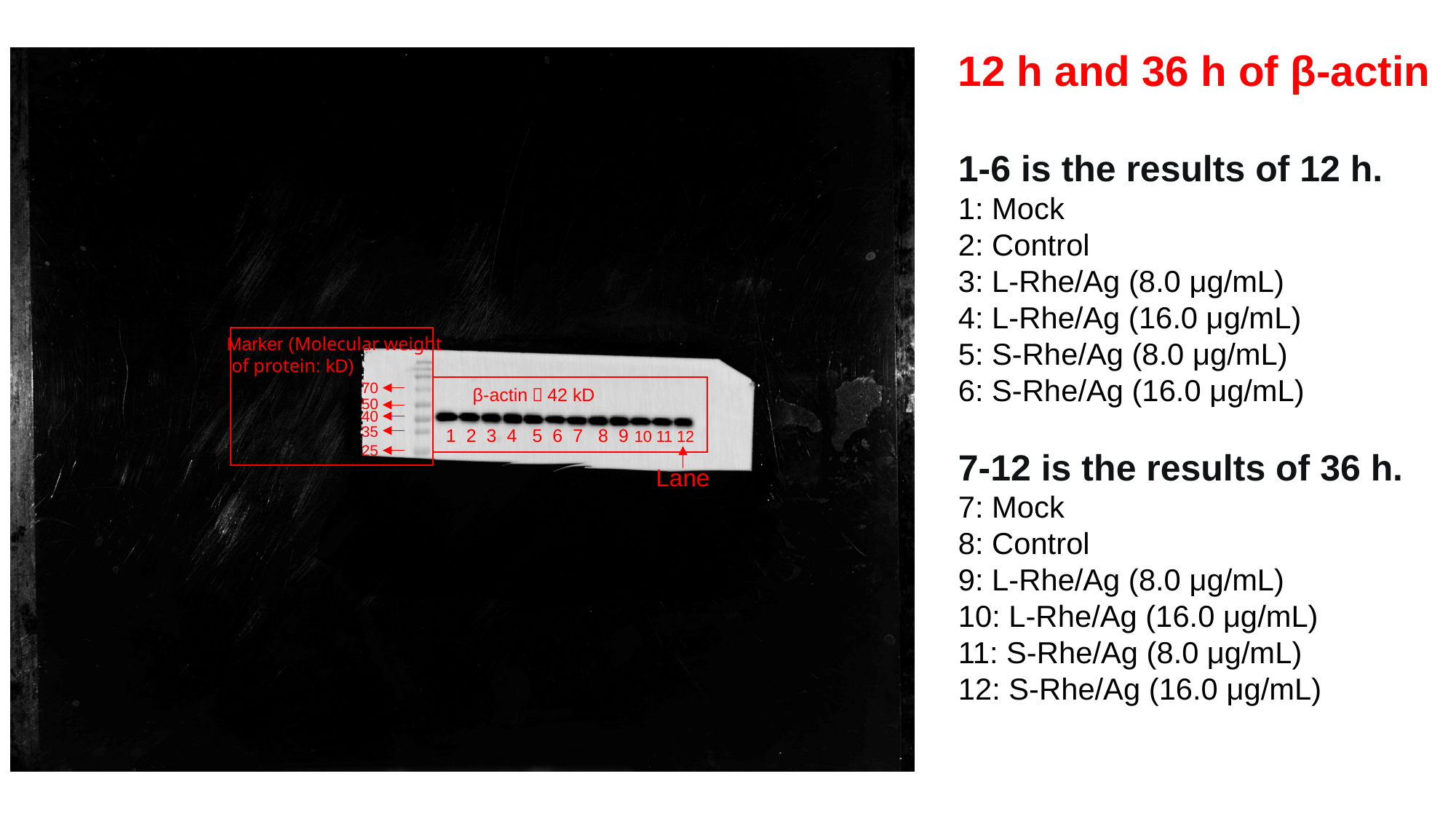

12 h and 36 h of β-actin
1-6 is the results of 12 h.
1: Mock
2: Control
3: L-Rhe/Ag (8.0 μg/mL)
4: L-Rhe/Ag (16.0 μg/mL)
5: S-Rhe/Ag (8.0 μg/mL)
6: S-Rhe/Ag (16.0 μg/mL)
7-12 is the results of 36 h.
7: Mock
8: Control
9: L-Rhe/Ag (8.0 μg/mL)
10: L-Rhe/Ag (16.0 μg/mL)
11: S-Rhe/Ag (8.0 μg/mL)
12: S-Rhe/Ag (16.0 μg/mL)
Marker (Molecular weight
 of protein: kD)
70
 β-actin：42 kD
50
40
35
1 2 3 4 5 6 7 8 9 10 11 12
25
Lane

## Slide 2
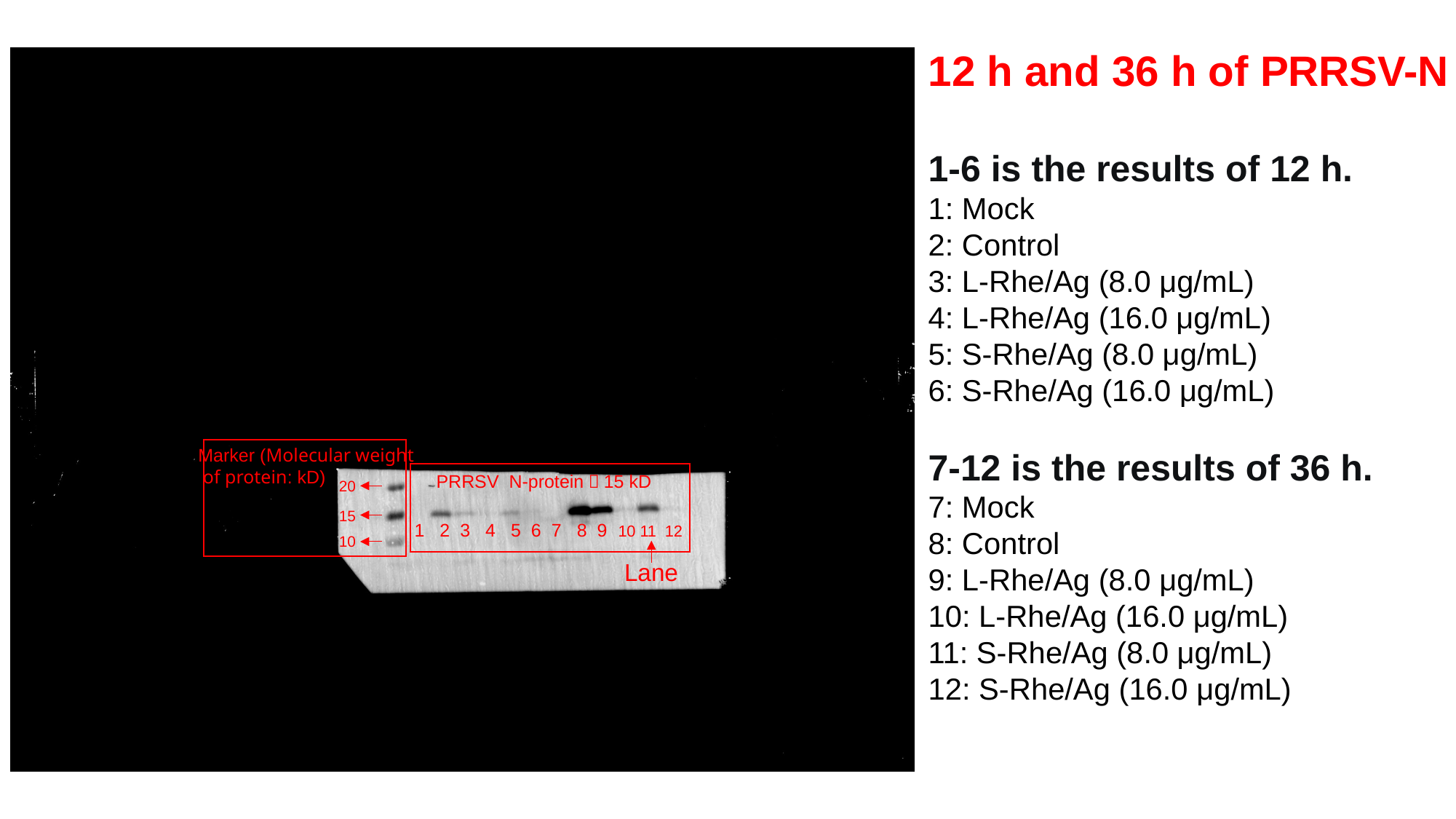

12 h and 36 h of PRRSV-N
1-6 is the results of 12 h.
1: Mock
2: Control
3: L-Rhe/Ag (8.0 μg/mL)
4: L-Rhe/Ag (16.0 μg/mL)
5: S-Rhe/Ag (8.0 μg/mL)
6: S-Rhe/Ag (16.0 μg/mL)
7-12 is the results of 36 h.
7: Mock
8: Control
9: L-Rhe/Ag (8.0 μg/mL)
10: L-Rhe/Ag (16.0 μg/mL)
11: S-Rhe/Ag (8.0 μg/mL)
12: S-Rhe/Ag (16.0 μg/mL)
Marker (Molecular weight
 of protein: kD)
PRRSV N-protein：15 kD
20
15
1 2 3 4 5 6 7 8 9 10 11 12
10
Lane

## Slide 3
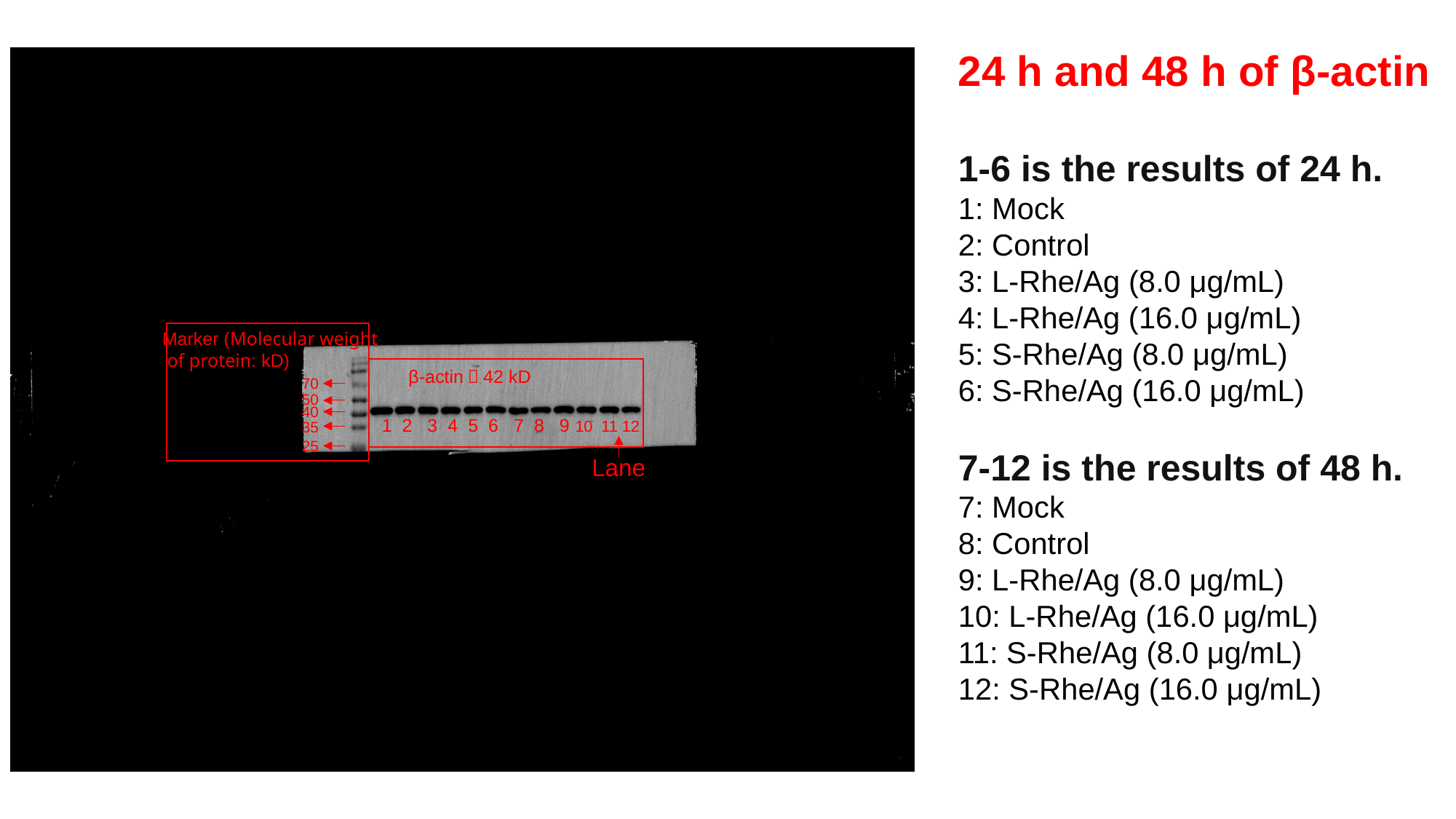

24 h and 48 h of β-actin
1-6 is the results of 24 h.
1: Mock
2: Control
3: L-Rhe/Ag (8.0 μg/mL)
4: L-Rhe/Ag (16.0 μg/mL)
5: S-Rhe/Ag (8.0 μg/mL)
6: S-Rhe/Ag (16.0 μg/mL)
7-12 is the results of 48 h.
7: Mock
8: Control
9: L-Rhe/Ag (8.0 μg/mL)
10: L-Rhe/Ag (16.0 μg/mL)
11: S-Rhe/Ag (8.0 μg/mL)
12: S-Rhe/Ag (16.0 μg/mL)
Marker (Molecular weight
 of protein: kD)
 β-actin：42 kD
70
50
40
1 2 3 4 5 6 7 8 9 10 11 12
35
25
Lane

## Slide 4
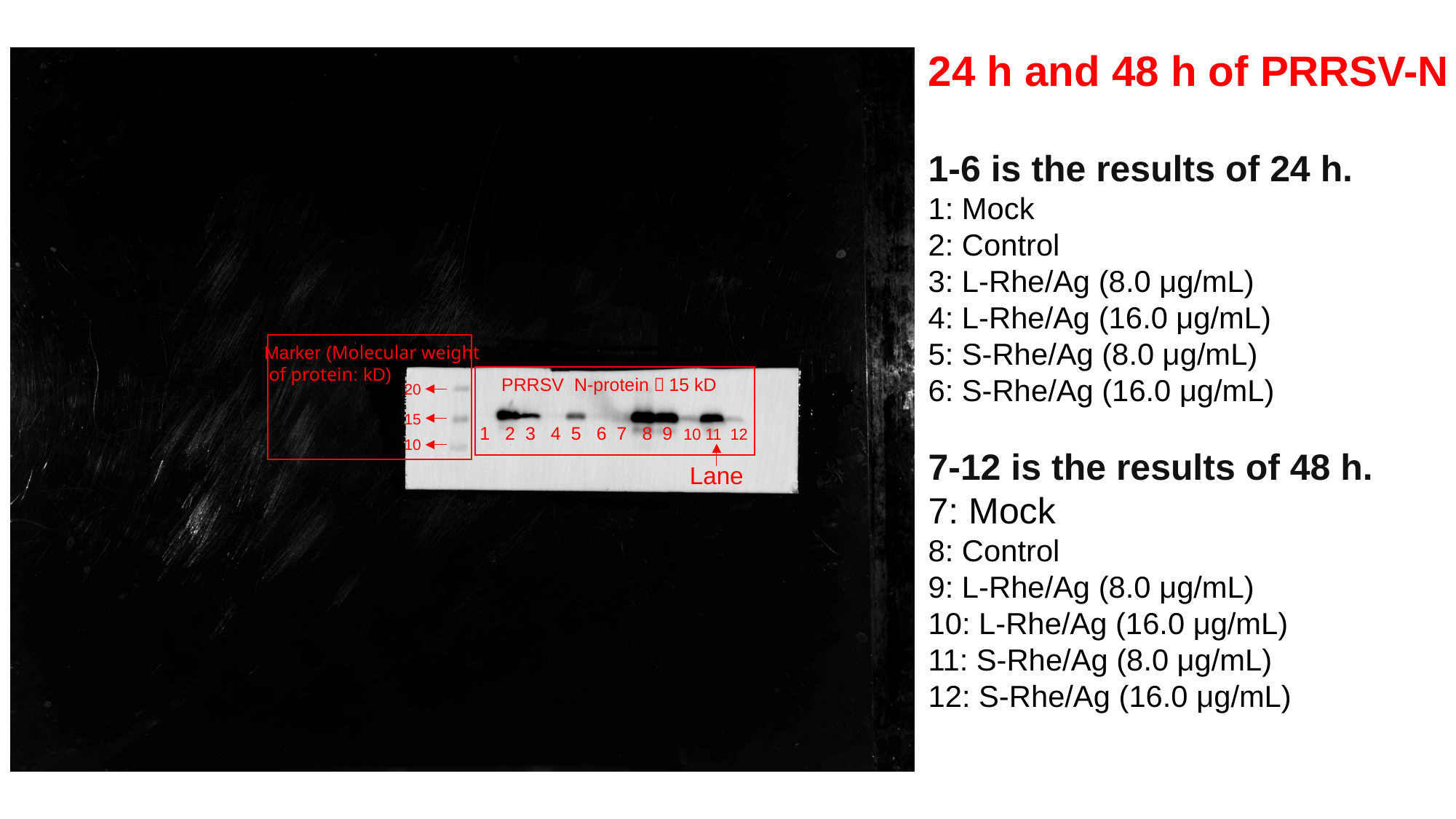

24 h and 48 h of PRRSV-N
1-6 is the results of 24 h.
1: Mock
2: Control
3: L-Rhe/Ag (8.0 μg/mL)
4: L-Rhe/Ag (16.0 μg/mL)
5: S-Rhe/Ag (8.0 μg/mL)
6: S-Rhe/Ag (16.0 μg/mL)
7-12 is the results of 48 h.
7: Mock
8: Control
9: L-Rhe/Ag (8.0 μg/mL)
10: L-Rhe/Ag (16.0 μg/mL)
11: S-Rhe/Ag (8.0 μg/mL)
12: S-Rhe/Ag (16.0 μg/mL)
Marker (Molecular weight
 of protein: kD)
PRRSV N-protein：15 kD
20
15
1 2 3 4 5 6 7 8 9 10 11 12
10
Lane
